# RNA Interference-Mediated Suppression of *GhSP* (*SELF-PRUNING*) Modulates the Plant Architecture of Transgenic Cotton in a Dose-Dependent Manner

**DOI:** 10.3390/biology14060601

**Published:** 2025-05-25

**Authors:** Yi Wang, Qinzhao Liu, Wanting Yu, Junmin Chen, Qingwei Suo, Zhong Chen, Jianyan Zeng, Aimin Liang, Jie Kong, Yuehua Xiao

**Affiliations:** 1 Chongqing Key Laboratory of Crop Molecular Improvement, College of Agronomy and Biotechnology, Southwest University, Chongqing 400715, China; norman@email.swu.edu.cn (Y.W.); 18338977812@139.com (Q.L.); yuwanting2024@163.com (W.Y.); 18188453237@163.com (J.C.); suoqw0302@email.swu.edu.cn (Q.S.); chenzhong77@163.com (Z.C.); zengjianyan@swu.edu.cn (J.Z.); liam2855@163.com (A.L.); 2 Bio-Resource Research and Utilization Joint Key Laboratory of Sichuan and Chongqing, Institute of Medicinal Plant Cultivation, Chongqing University of Chinese Medicine, Chongqing 402760, China; 3 Xinjiang Key Laboratory of Cotton Genetic Improvement and Intelligent Production, Cotton Research Institute, Xinjiang Academy of Agricultural Sciences, Urumqi 830091, China; kongjie.258@163.com

**Keywords:** cotton architecture, *SELF-PRUNING*, dose dependent, RNA interference

## Abstract

Cotton exhibits indeterminate growth potential at its apical meristem. In field cultivation, it is often necessary to restrict plant height by the foliar application of plant growth regulators or artificial topping. The genetic engineering of cotton architecture offers an efficient, environmentally friendly, and low-cost alternative to current field management. Our study aimed to improve the plant architecture of transgenic cotton by the suppression of *GhSP*, a key flowering repressor, via the RNA interference method. Sixteen independent transgenic lines were generated and classified as mildly, moderately, and severely suppressed, according to *GhSP* expression levels. Field evaluation revealed the dose-dependent effects of *GhSP* silencing on plant height. The mildly suppressed line *GhSPi*-#5 exhibited a semi-dwarf phenotype of approximately 70~100 cm in height. Negative phenotypes, including excessive dwarf plant architecture and inferior fiber quality and yield traits, were observed in severely *GhSP*-suppressed transgenic lines. Notably, the mild silencing of *GhSP* in *GhSPi*-#5 did not negatively affect leaf and flower organ growth, pollen fertility, major agronomic traits, or fiber quality compared with the wild type. These observations demonstrate the feasibility of manipulating the architecture of transgenic cotton via *GhSP* silencing.

## Simple Summary

To improve cotton architecture for field cultivation, this study employed RNA interference (RNAi) to achieve the graded suppression of *GhSP* (a key flowering repressor). Field trials revealed the dose-dependent effect of *GhSP* silencing on the determinate growth and final plant height of transgenic cotton. A mild suppression of *GhSP* in line *GhSPi*-#5 led to a semi-dwarf phenotype (70~100 cm) with preserved agronomic traits, which was ideal for cotton production compared to the indeterminate growth of the wild type and excessive dwarfism and compromised fiber quality in severely *GhSP*-suppressed cotton. These results demonstrate that the precise manipulation of *GhSP* expression enables targeted improvement in cotton plant architecture.

## 1. Introduction

Crop architecture comprises plant height, branching patterns, and leaf morphology including shape and size characteristics, with the height and branching features serving as the primary determinants of the architecture formation. Excessively tall plant height increases susceptibility to lodging, which impedes mechanized harvesting, crop yields, and quality formation, whereas extreme dwarfism in the morphological structure frequently diminishes yield potential. An ideal plant architecture is critical for achieving equilibrium between the crop yield potential and lodging resistance in agricultural systems [1,2]. Cotton (*Gossypium* spp.) constitutes a strategic economic crop, producing the majority of the natural fiber for the global textile industry. Cotton exhibits a monopodial primary axis that maintains indeterminate apical dominance under non-manipulated developmental conditions [3]. Current agronomic practices predominantly employ the foliar application of plant growth regulators (such as mepiquat chloride) and artificial topping to control cotton vertical growth [4,5]. These methods impose significant labor input and the potential risk of xenobiotic contamination from agrochemical leaching. To address the dual imperatives of fiber yield optimization and mechanized harvesting efficiency, breeding programs are increasingly prioritizing the development of semi-dwarf cotton with compact architecture [6,7,8].

The precise regulation and dynamic interplay between florigen and anti-florigen is critical to determine the flowering and apical growth habitat and, consequently, plant architecture [9]. Flowering Locus T (FT) is the first identified florigen from Arabidopsis. FT and its homologs (such as SINGLE FLOWER TRUSS or SFT in tomato), synthesized in leaves, are transported to the apical meristem and promote flowering. Anti-florigens comprise the *CEN/TFL1/SP* (*CETS*) gene family, including *CENTRORADIALIS* (*CEN*) from Antirrhinum, *TERMINAL FLOWER1* (*TFL1*) from Arabidopsis, *SELF-PRUNING* (*SP*) from tomato, and their conserved homologs in taller plants [10,11,12,13]. These anti-florigens primarily function antagonistically with florigens to inhibit the transition to flowering and maintain the indeterminate activity of meristems [14,15]. The SFT (florigen)/SP (anti-florigen) balance model posits that an elevated SFT/SP ratio within meristems drives determinate growth, ultimately leading to apical termination, whereas a reduced ratio sustains indeterminate growth [3,16].

In tomato, mutations in the *SP* gene induce precocious flowering, progressively reducing leaf numbers in sympodial units and terminating shoots with two consecutive inflorescences, thereby driving the shift from indeterminate to determinate growth [11,17]. The *SP* mutation has emerged as a pivotal target for crop genetic improvement, revolutionizing tomato breeding. Cultivars harboring *SP* mutations are recognized as cornerstone genetic traits in modern agriculture due to their controlled growth habit and stable yield [10,11,18]. Combinatorial screening with other floral transition pathway genes has further optimized tomato productivity [19]. This strategy has been successfully extended to crops including soybean [20], grapes [21], barley [22], roses [23], and strawberries [23], demonstrating the universal regulatory role of SP in plant architecture modulation.

In recent years, the cotton ortholog of the SELF-PRUNING (GhSP/GhCEN/GhTFL1) gene has emerged as a research focus for its precise regulation of plant architectural development. The virus-induced gene silencing (VIGS) of *GhSP* triggers precocious flowering and premature growth cessation, forming terminal flowers on dwarfed main stems and shortened lateral branches [14,15,16,24,25]. The complete knockout of *GhSP* via CRISPR/Cas9 results in extreme negative traits, precluding their utility in breeding applications [8,26,27]. Remarkably, an L86P substitution in GhTFL1—a targeted mutation weakening (but not abolishing) protein interaction capacity—achieved partial loss of function, generating elite germplasm with optimal plant architecture with moderate height, compact fruiting branches, and a shortened reproductive phase [8].

Notably, cotton GhSP exerts anti-florigen functions with a dose effect because the varied activities and mutant copies in the four homologous alleles result in different phenotypic variations in flowering and plant architecture [4,8]. Nevertheless, there currently lacks detailed analysis of the dose relationship between *GhSP* expression levels and phenotypic outcomes, and the ideal levels of *GhSP* expression for balancing plant architecture and yield remain to be elucidated. To address these gaps, this study employed RNA interference (RNAi) technology to generate transgenic cotton lines with gradually lowered *GhSP* expression levels. We observed that the mildly suppressed *GhSP* RNAi line demonstrated self-pruning capacity while retaining core agronomic traits comparable to the wild type. These findings underscore that the precise optimization of *GhSP* expression levels, based on this line’s dose-dependent regulatory mechanism, enables simultaneous improvement in cotton architecture (from semi-dwarf to extreme dwarfing gradients) and coordinated yield traits. This approach establishes a novel molecular breeding strategy for the synergistic enhancement of plant architecture and yield traits in crops.

## 2. Materials and Methods

### 2.1. Plant Material and Growth Conditions

The tetraploid upland cotton (*G. hirsutum* L., AD genome, 2n = 4x = 52) cultivar Jimian No.14 was used for cotton transformation. Cotton seedlings were grown in a climate-controlled greenhouse with a 16 h/8 h (light/dark) schedule and the temperature kept at 26–30 °C. Field-grown cotton plants in a net house in Chongqing, China, were subjected to architectural characterization during the growing season, with the concomitant collection of fiber and ovule specimens from these cultivated populations.

### 2.2. Vector Construction and Plant Transformation

An intron-containing hairpin RNA construct of the cotton *SP* gene (*GhSP*-RNAi) was amplified from cotton genomic DNA as previously described [28]. The 20 µL PCR mixture included 50 ng of cotton genomic DNA, a 400 nM flanking primer (5′-GATTCAGATCTAGGGCAAAC-3′), a 40 nM bridge primer (5′-CCTTGGCATTTC GTAGTTCACAACAGATGCCACATTTGGT-3′), and 10 µL of 2 × PrimeSTAR^®^ Max Premix (Takara, Dalian, China). The PCR thermocycling parameters were as follows: 95 °C for 5 min, followed by 35 cycles of 95 °C for 30 s, 56 °C for 30 s, and 72 °C for 30 s and a final extension of 10 min at 72 °C. The *GhSP*-RNAi fragment was cloned into the pGEM-T Easy Vector (Promega, Madison, WI, USA) and sequenced and then inserted into a modified pBI121 vector p5 containing selection marker *GUS* and *NPTII* genes [29]. The Agrobacterium-mediated transformation of cotton was conducted according to a previously established protocol with minor modifications [29]. Cotton calluses were selected on an MS medium containing 50 mg/L of kanamycin with subculturing every 15 days. The regenerated plants (T_0_) were verified by PCR amplification of the transgene. The primers are listed in Table S1.

T_1_ plants generated from the selfing seeds of T_0_ transformants were selfed to produce T_2_ seeds, which were sown to form T_2_ lines. For each transformant, homozygous T_2_ lines (without non-transgenic segregants) were selected for phenotypic observation and molecular characterization.

### 2.3. RNA Isolation and RT-qPCR

The shoot tips of transgenic and wild-type seedlings at 14 days after sowing (DAS) were ground in liquid nitrogen, and total RNA was extracted using a Plant Total RNA Extraction kit (Tiangen Biotech, Beijing, China) according to the manufacturer’s instructions. First-strand cDNA was synthesized from 1 µg of total RNA using a RT SuperMix Kit (Vazyme, R233-01, Nanjing, China) with a gDNA wiper to eliminate genomic DNA contamination. RT-qPCR amplifications were performed with ChamQ Universal SYBR qPCR Master Mix (Vazyme, Nanjing, China) on the CFX96 Real-Time System (Bio-Rad, Hercules, CA, USA). The thermocycling parameters were as follows: 95 °C for 3 min, followed by 40 cycles of 95 °C for 5 s and 57 °C for 20 s, and a standard melting curve program was added to monitor PCR specificity. Each analysis was repeated with three biological replicates. The primers are listed in Table S1. The *GhACT* gene served as an internal control.

### 2.4. Chlorophyll Extraction and Quantification

Chlorophyll extraction and quantification in leaves were performed by a modified protocol [30]. In brief, the target leaf samples were thoroughly rinsed and blotted dry, the leaf vein was removed, and the leaf was cut into strips approximately 2 mm wide. Next, 0.5 g of the prepared leaf strips was transferred to a test tube, 10 mL of 80% acetone was added to fully submerge the tissue, and the tube was sealed tightly. The leaf strips were incubated in a darkroom for a minimum of 24 h, with the tube shaken 2–3 times during the extraction period until the strips became completely bleached. The absorbances at 646 and 663 nm were determined using a spectrophotometer (Varioskan LUX multimode microplate reader, Thermo Fisher Scientific, Waltham, MA, USA). Three biological replicates were used for each sample. The chlorophyll contents were represented by the following formulas:

$$C_{A}=\frac{(12.21\times A_{663}-2.81\times A_{646})\times V}{1000\times W}$$

$$C_{B}=\frac{(20.13\times A_{646}-5.03\times A_{663})\times V}{1000\times W}$$

where *V* represents the volume of the extraction solution, and *W* represents the weight of the sample in grams.

### 2.5. Net Photosynthetic Rates

Net photosynthetic rates were measured in three fully expanded leaves per cotton plant using an LI-6400/XT Portable Photosynthesis System (LI-COR, Lincoln, NE, USA). Measurements were conducted between 10:00 a.m. and 12:00 p.m. under clear-sky conditions, following established protocols as previously described [31].

### 2.6. Observation of Pollen Morphology

For the purpose of analyzing pollen fertility, pollen grains at the stage of anther dehiscence were liberated and subsequently stained with an I_2_-KI solution. Following this, photographs were taken using a Zeiss Axio Imager Z2 microscope (Zeiss, Oberkochen, Germany). In addition, the morphological characteristics of the anther and pollen grains were observed and photographed using a scanning electron microscope (SEM) platform (SU3500, Hitachi, Tokyo, Japan), operated at an accelerating voltage of 3 kV.

### 2.7. Histological Sectioning

Histological sections of the shoot tips were prepared as described previously [32]. In brief, shoot apices were cut from cotton seedings at 14 DAS and immediately immersed in a 50% FAA fixative solution (absolute ethanol: formaldehyde: acetic acid, 10:2:1). Dehydration was performed in a graded ethanol series (30%, 50%, 70%, 95%, and 100%, all *v*/*v*), and then the ethanol was gradually replaced with xylene. The tissues were embedded in paraffin wax and sliced into sections with a thickness of 10 µm using a Leica RM2235 Rotary Microtome (Leica, Wetzlar, Germany). Toluidine blue (1% *w*/*v*) was used to stain the sections. A Zeiss Axio Imager Z2 microscope (Zeiss, Oberkochen, Germany) was used to image the samples under brightfield illumination.

For the microscopic observation of the mature fibers, naturally opened bolls were harvested on the same day. Only the six cottonseeds in the middle of the locule were collected for microscopic analysis. The fibers from the middle of the cottonseed were fixed for transverse sectioning as described in previous research [33]. Briefly, the 1 cm segments were excised from the mid-region of the bundled fibers, dehydrated in a graded ethanol series, embedded in paraffin, and sectioned into 10 µm-thick slices using a rotary microtome (Leica RM2235, Wetzlar, Germany). The sections were stained with fast green (1% *w*/*v*), then observed and photographed under an optical microscope (Zeiss Axio Imager Z2, Zeiss, Oberkochen, Germany). The wall thickness of fiber transverse sections were measured in the collected images using ImageJ 2 (http://imagej.net/Fiji, accessed on 22 May 2025).

### 2.8. Cotton Fiber Quality Assessment

The naturally matured cotton of each line was harvested in late August for fiber yield trait evaluation. After drying and ginning using a roller gin (SY-20, Jianghe Machinery Plant, Xinxiang, Henan, China), the fibers and cottonseeds were separately weighed to determine lint percentages. To evaluate fiber quality, a minimum of 15 g of fibers was collected per sample. Fiber quality traits (including fiber length, the uniformity index, strength, the micronaire value, and the elongation ratio) were analyzed using a High-Volume Instrument (HVI) test system (HFT 9000, Uster Technologies AG, Uster, Switzerland), in accordance with Chinese National Standard GB/T 20392-2006 [34], at the Center for Cotton Fiber Quality Inspection and Testing at the Chinese Ministry of Agriculture (Anyang, Henan, China). Three biological replicates were analyzed per sample.

## 3. Results

### 3.1. Down-Regulation of GhSP in Cotton

**Figure 1:**
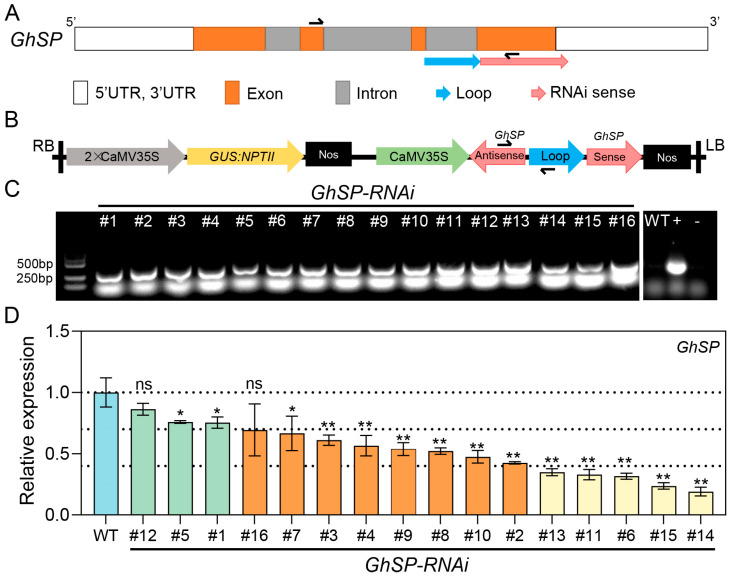
Suppressing the expression of *GhSP* in cotton via RNA interference. (**A**) Schematic representation of the *GhSP* gene structure. The positions of the *GhSP* specific primers for RT-qPCR are indicated by the black arrows. (**B**) Schematic diagram of the T-DNA construct for the RNAi vector targeting the *GhSP* gene in transgenic cotton. The transgene specific primers are indicated by the black arrows. CaMV35S, cauliflower mosaic virus 35S promoter; 2 × CaMV35S, two CaMV35Ss in series; Nos, the terminator region of *A. tumefaciens* nopaline synthase gene; *NPTII*, the neomycin phosphotransferase gene; *GUS*, the β-glucuronidase gene. (**C**) PCR amplification validation of transgenic cotton plants (#1~16). Negative controls included WT and ddH_2_O (−), and the positive control (+) was the RNAi vector CaMV35S:*GhSP*-RNAi. (**D**) Relative expression levels of *GhSP* in T_2_ transgenic cotton plants. RNA was isolated from the shoot apex (14 DAS) of transgenic cotton plants and the wild type. Statistical significance was determined by Student’s *t*-test (*n* = 3). *, *p* < 0.05, **, *p* < 0.01; ns, not significant.

To avoid the severe dwarfism phenotype resulting from *GhSP* knockout in cotton (*G. hirsutum*) [27], RNA interference (RNAi) was employed to modulate *GhSP* expression levels and improve the cotton architecture. Accordingly, an shRNA-based interference element was designed using the third intron of the *GhSP* gene as the loop and the fourth exon as the sense strand (Figure 1A). The interference element was driven by a cauliflower mosaic virus (CaMV) 35S promoter to assemble the RNAi vector CaMV35S:*GhSP*-RNAi (Figure 1B). A total of 16 independent transgenic CaMV35S:*GhSP*-RNAi (*GhSPi*) cotton lines were obtained through cotton genetic transformation and were further confirmed with PCR analysis (Figure 1C). RT-qPCR analysis revealed the different-level suppression of *GhSP* gene expression in transgenic lines compared to the wild type (Figure 1D). These transgenic cotton lines were categorized into three groups based on the degree of *GhSP* expression suppression, i.e., mildly suppressed (the *GhSP* expression level was decreased by less than 30%; *GhSPi*-#1, -#5, and -#12), moderately suppressed (decreased by 30–60%; *GhSPi*-#2, -#3, -#4, -#7, -#8, -#9, -#10, and -#16), and severely suppressed (over 60%; *GhSPi*-#6, -#11, -#13, -#14, and -#15). Representative transformants *GhSPi*-#5 (mild), *GhSPi*-#8 (moderate), and *GhSPi*-#14 (severe) were selected for subsequent phenotypic analyses (Figure 1D).

### 3.2. Silencing GhSP Induces Determinate Main Stems in Transgenic Cotton in a Dose-Dependent Manner

**Figure 2:**
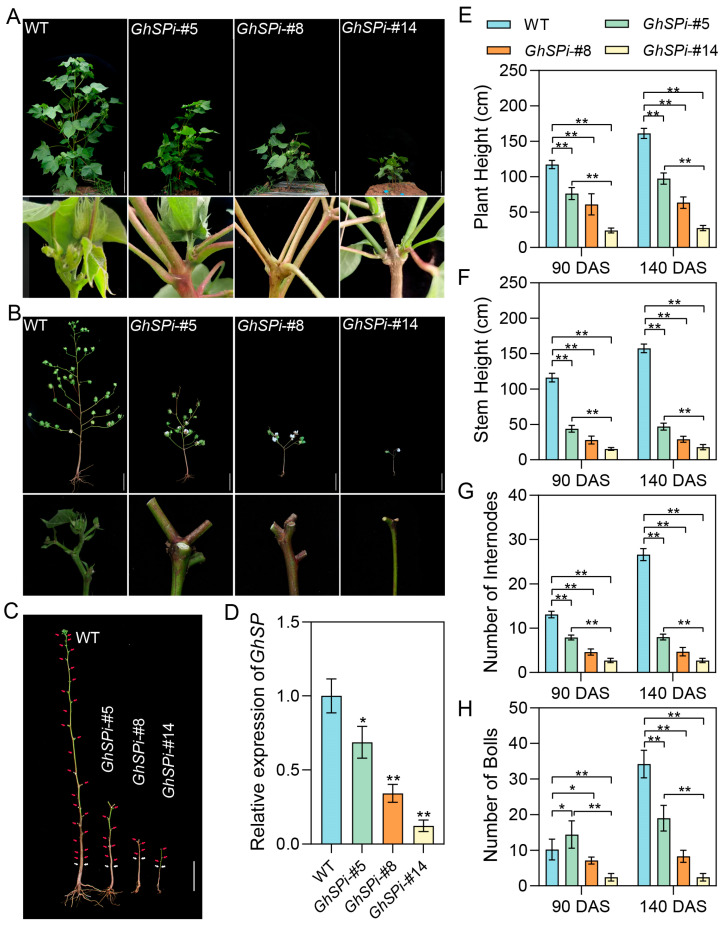
Plant architecture of *GhSPi* transgenic cotton. Morphological comparison of plant architecture between *GhSPi* transgenic cotton and the wild type at 90 DAS (**A**) and 140 DAS (**B**). (**C**) Main stems of *GhSPi* transgenic and wild-type cotton at 140 DAS. White arrows designate the cotyledon location; red arrows mark the node position. (**D**) RT-qPCR analysis of *GhSP* expression in *GhSPi* transgenic cotton and the wild type at 14 DAS. Statistical significance was determined by Student’s *t*-test (*n* = 3). *, *p* < 0.05, **, *p* < 0.01. (**E**–**H**) Quantitative assessment of plant height (**E**), stem height (**F**), internode number (**G**), and boll number (**H**) in *GhSPi* transgenic lines and the wild type. Scale bars, 20 cm. Statistical significance was determined by Student’s *t*-test (*n* = 10). *, *p* < 0.05, **, *p* < 0.01.

To investigate the impact of varying *GhSP* silencing levels on cotton architecture, the T_2_ generations of *GhSPi*-#5, *GhSPi*-#8, and *GhSPi*-#14 were sown in a field. All the *GhSPi* transgenic cotton lines exhibited self-pruning at 90 days after sowing (DAS), showing graded architectural alterations. *GhSPi*-#5 displayed a semi-dwarf architecture (70~100 cm), *GhSPi*-#8 showed a dwarf phenotype (50~70 cm), and *GhSPi*-#14 demonstrated extreme dwarfism (20~40 cm) consistent with the *GhSP* knockout line (Figure 2A) [27]. At 140 days after sowing (boll maturation stage), *GhSPi*-#5, *GhSPi*-#8, and *GhSPi*-#14 still maintained the self-pruning architecture (Figure 2B,C). Quantitative analyses revealed that *GhSPi* transgenic cotton lines exhibited significant reductions in plant height, main stem height, and the internode number compared to the wild type. These architectural parameters showed strong positive correlations with *GhSP* transcript levels (Figure 2D–G). The boll count, a critical determinant of cotton yield, is intricately modulated by plant architecture. Statistical analysis revealed that the number of cotton bolls in *GhSP* transgenic lines was significantly reduced compared to the wild type, except for an increase observed in *GhSPi*-#5 at 90 DAS. Like plant height, main stem height, and the internode number, the boll count of *GhSPi* transgenic cotton displayed a positive correlation with *GhSP* expression levels (Figure 2H). These results demonstrated that *GhSP* expression levels exhibited the dose-dependent regulation of cotton architecture. Notably, *GhSPi*-#5, with the mild down-regulation of *GhSP* expression, achieved self-pruning architecture while preventing the extreme phenotypic aberrations observed in *GhSPi*-#14 (Figure 2).

The activity of shoot apical meristems (SAM) is coordinately regulated by complex genetic networks and influenced by internal/external environments, ultimately determining plant architecture and the flowering time. To further investigate the impact of altered *GhSP* expression levels on apical meristem differentiation, histological analyses were performed on shoot apices at 14 DAS. Comparative observations revealed that *GhSPi*-#5 exhibited wild type-like apical bud morphology with sustained growth potential, whereas *GhSPi*-#14 displayed complete conversion to flower bud and the loss of the upward differentiation capacity (Figure S1A). These findings suggested that the suppression of *GhSP* expression induced apical meristem ablation, leading to determinate growth, while the mild suppression of *GhSP* levels delayed this developmental switch. GhSP interacts with GhFD to mediate the antagonistic suppression of florigen GhFT activity, thereby establishing the transcriptional repression of downstream inflorescence meristem regulatory genes such as *APETALA1* (*GhAP1*) [6]. RT-qPCR analysis revealed up-regulated *GhAP1* expression in the shoot apices of the *GhSPi*-#5, *GhSPi* -#8, and *GhSPi* -#14 lines at 14 DAS, showing a negative correlation with *GhSP* expression levels (Figure S1B). This transcriptional repressive relationship suggested that *GhSP*-mediated *GhAP1* suppression was also dose-dependent.

### 3.3. Effects of GhSP Suppression on Leaf and Floral Organ Development

**Figure 3:**
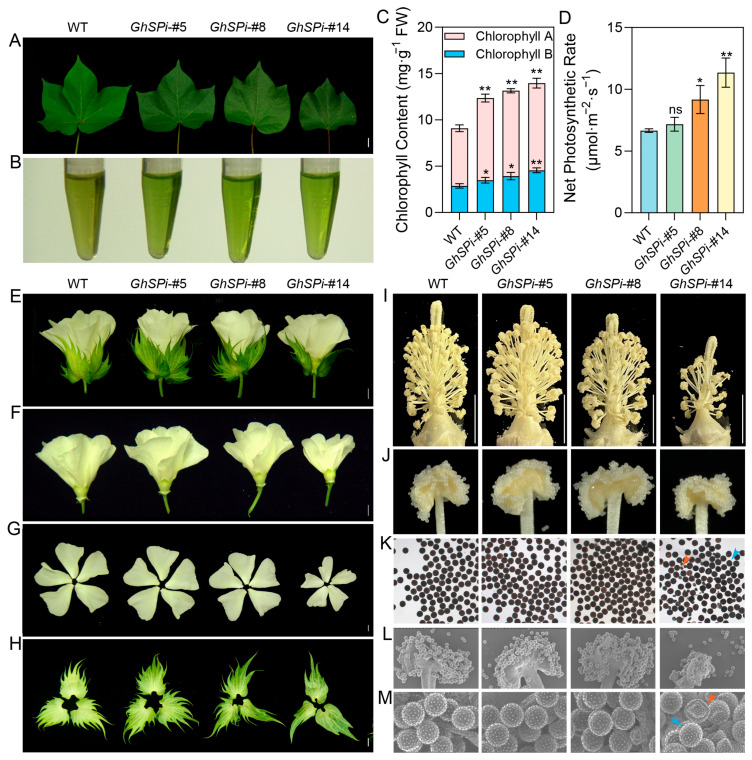
Leaf, flower, and anther phenotypes of *GhSPi* transgenic cotton. (**A**) Leaves of *GhSPi* transgenic cotton and the wild type. Scale bars, 20 mm. Extraction (**B**) and measurement (**C**) of chlorophyll content in the leaves of *GhSPi* transgenic cotton and the wild type. (**D**) Net photosynthetic rate of *GhSPi* transgenic cotton and wild-type leaves. Whole flower (**E**), bract-peeled flower (**F**), petals (**G**), and bracts (**H**) of *GhSPi* transgenic cotton and the wild type. Scale bars, 10 mm. (**I**,**J**) The anthers from day 0 post-anthesis of *GhSPi* transgenic cotton and the wild type. Scale bars, 10 mm. Comparative analysis of pollen morphology in *GhSPi* transgenic cotton and the wild type via I_2_-KI staining (**K**) and scanning electron microscopy (**L**,**M**). Blue arrows denote normal pollen grains; red arrows highlight shrunken (abortive) pollen grains. Scale bars, 10 mm. Significant differences between *GhSPi* transgenic plants and the wild-type control in C and D were determined by Student’s *t*-test. *, *p* < 0.05; **, *p* < 0.01; ns, not significant, *n* = 3.

Leaves serve as the primary site for photosynthesis in plants. Compared to the wild type, the *GhSPi*-#14 line exhibited a smaller leaf size and darker coloration, while *GhSPi*-#5 displayed leaf dimensions comparable to the wild type (Figure 3A). Chlorophyll content analysis revealed a significant increase in *GhSPi* transgenic cotton relative to the WT (Figure 3A,B). Furthermore, the net photosynthetic rate was enhanced in *GhSPi* transgenic lines compared to the wild type, except for *GhSPi*-#5 (Figure 3D). Observations of the floral organs on the day of anthesis demonstrated that the severely dwarfed *GhSPi*-#14 line developed smaller flowers and petals but produced larger, darker-colored bracts, while the *GhSPi*-#5 (semi-dwarf) line showed no significant differences in floral organ development compared to the wild type, indicating that the appropriate suppression of *GhSP* expression does not critically affect these traits (Figure 3E–H). Pollen fertility and viability are fundamental to plant reproductive success and are critically linked to cotton yield. *GhSPi*-#14 exhibited shortened stigmas and reduced anther numbers, whereas the *GhSPi*-#5 and *GhSPi*-#8 lines showed stigma and anther characteristics comparable to those of the WT (Figure 3I,J). Microscopic analysis revealed no significant differences in pollen fertility among *GhSPi*-#5, *GhSPi*-#8, and the wild type, whereas *GhSPi*-#14 displayed partially shrunken pollen grains (approximately 16%) (Figure 3K). Further scanning electron microscopy (SEM) observation of pollen morphology confirmed these findings. These results imply that the extreme suppression of *GhSP* (e.g., *GhSPi*-#14) induces leaf size reduction, floral organ malformation, and partial pollen sterility, whereas the mild down-regulation of *GhSP* expression (e.g., *GhSPi*-#5) maintains normal leaf and floral development, preserves pollen fertility, and rescues the associated negative traits caused by the excessive suppression of *GhSP* expression.

### 3.4. Mild Silencing of GhSP Preserves Key Agronomic Traits in Cotton

**Figure 4:**
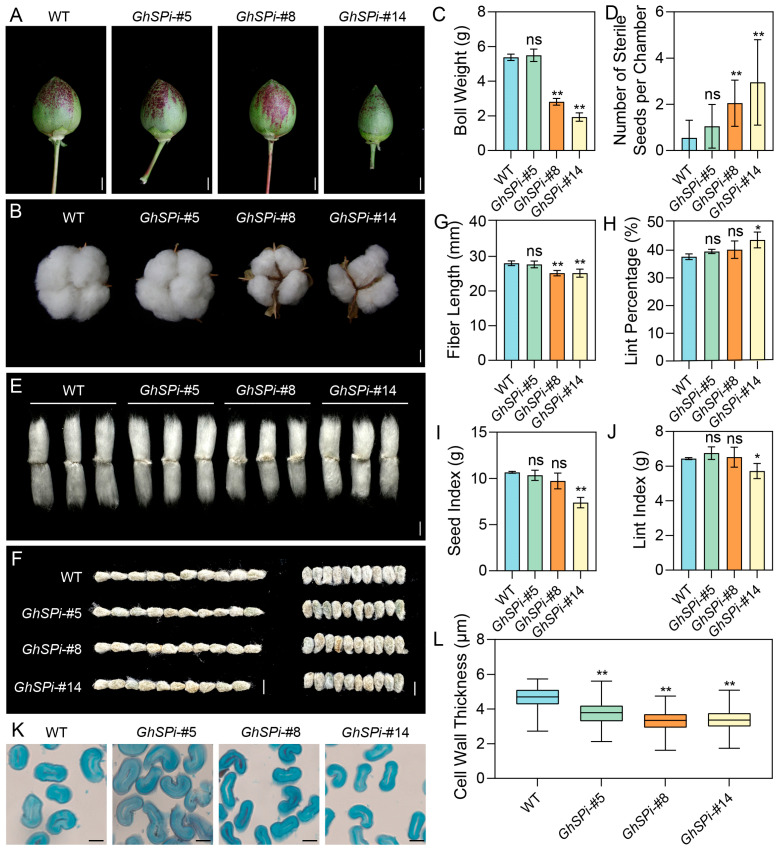
Several agronomic traits related to yield in *GhSPi* transgenic cotton. Immature (at 30 DPA, (**A**)) and mature (**B**) cotton bolls from transgenic and wild-type plants. Scale bars, 10 mm. (**C**) Weight of individual mature cotton bolls (*n* = 18). (**D**) Number of sterile seeds in individual chambers (*n* = 20). Mature fibers (**E**) and seeds (**F**) of *GhSPi* transgenic plants and the wild type. Scale bars, 10 mm. Measurement and statistical comparison of fiber length (**G**), lint percentage (**H**), the seed index (**I**), and the lint index (**J**) were conducted between *GhSPi* transgenic cotton lines and the wild type (*n* = 3). (**K**) Microscopic section observation of mature fibers. Scale bars, 10 µm. (**L**) Statistical analysis of mature fiber cell wall thickness (*n* > 150). Student’s *t*-test was used for statistical analyses, *, *p* < 0.05, **, *p* < 0.01; ns, not significant.

To identify the effects of plant architecture alterations caused by *GhSP* down-regulation on cotton yield and the key agronomic traits of the *GhSPi* lines and the wild type were evaluated. The bolls of the extremely dwarfed line *GhSPi*-#14 were markedly smaller than those of the WT, whereas the semi-dwarfed line *GhSPi*-#5 exhibited boll dimensions comparable to those of the WT (Figure 4A,B). Quantitative analysis of boll weight revealed that *GhSPi*-#5 exhibited no significant difference compared to the WT, while *GhSPi*-#8 and *GhSPi*-#14 showed significant reductions (Figure 4C). The primary cause of this outcome likely stems from a low proportion of sterile seeds in *GhSPi*-#5, comparable to the wild type, whereas *GhSPi*-#8 and *GhSPi*-#14 harbored substantially more sterile seeds, thereby limiting their boll weight development (Figure 4D). Additionally, we observed some conjoined bolls in the *GhSPi* lines, with the frequency of this trait increasing as *GhSP* expression levels decreased (Figure S2). Fiber length serves as a critical parameter for evaluating cotton fiber quality. Our study demonstrated that fibers from *GhSPi*-#8 and *GhSPi*-#14 were significantly shortened compared to the wild type, whereas no statistically significant difference was observed in *GhSPi*-#5 (Figure 4E,G). Additionally, *GhSPi*-#14 exhibited a markedly reduced seed size, while both *GhSPi*-#5 and *GhSPi*-#8 showed no significant variation relative to the wild type (Figure 4F). Analysis of several yield-related traits revealed a marked increase in lint percentage for *GhSPi*-#14; however, its seed index and lint index showed significant reductions, collectively reflecting an overall yield loss. In contrast, *GhSPi*-#5 and *GhSPi*-#8 exhibited no statistically significant differences in these parameters compared to the wild type (Figure 4H–J). Further fiber quality analysis revealed that *GhSPi*-#5 displayed no significant differences in fiber properties compared to the wild type, except for a reduced micronaire value.While the excessive suppression of *GhSP* expression (*GhSPi*-#8 and *GhSPi*-#4) led to shortened fiber length, a decreased uniformity index, and reduced micronaire values, which collectively indicate inferior fiber quality (Table S2). Additionally, a cross-sectional analysis of fibers demonstrated that *GhSPi* transgenic cotton exhibited reduced fiber cell wall thickness compared to wild-type plants, with this phenotype being more pronounced in *GhSPi*-#8 and *GhSPi*-#14 (Figure 4K,L).

Taken together, these results suggest that the mild suppression of *GhSP* expression (e.g., in *GhSPi*-#5) improves cotton architecture without significant fiber quality and yield penalties. Conversely, the excessive inhibition of *GhSP* causes significant declines in both fiber quality and yield.

## 4. Discussion

The florigen (FT/SFT) and anti-florigen (TFL1/SP) systems collaboratively regulate vegetative and reproductive growth, determining plant architecture. This regulatory module has been harnessed in crop improvement to enhance yields across species. In tomato, *SP* mutants are recognized as one of the most transformative genetic traits in modern agriculture [11]. By leveraging combinations of selected mutations involving various components of the flowering pathway, tomato productivity has been further optimized [19]. For instance, tomato plants heterozygous for a loss-of-function allele of SFT exhibit up to a 60% increase in yield [35]. In cotton, cotton leaf crumple virus (CLCrV)-mediated *GhFT*/*SFT* over-expression decouples flowering from photoperiodic control, inducing premature flowering while retaining the indeterminate growth habit of main stems. Conversely, tobacco rattle virus (TRV)-driven *GhFT*/*SFT* silencing delays maturation and amplifies indeterminacy [3,15,16]. The over-expression of *GhSP* suppresses the vegetative-to-reproductive transition in branches, while its silencing triggers determinate growth, with terminal flowers on the main stem and shorter lateral branches [14,16,24]. These findings underscore the significant potential of the florigen/anti-florigen system in cotton breeding improvement. For example, the GhTFL1^L86P^ mutation, which weakens GhTFL1’s protein interaction capacity, produces semi-dwarf, compact cotton plants [8]. Our research employed RNA interference technology to generate heritable transgenic cotton with stepwise changes in *GhSP* expression levels. Among these, cotton plants with severely suppressed *GhSP* expression levels exhibited extreme dwarfism, similar to that of plants with CRISPR/Cas9-knockout *GhSP* [27]. In contrast, the *GhSPi*-#5 line with mild *GhSP* suppression achieved a semi-dwarf phenotype (Figure 1). The expression levels of *GhSP* exhibited negative correlation with cotton height, the internode number, boll weight, etc., confirming that *GhSP* exerts its functions in a dose-dependent manner.

Unlike the urgent demand for lodging resistance traits in crops such as rice and wheat, the pursuit of plant architecture in cotton breeding is primarily driven by the need to adapt to high-density planting and mechanical harvesting. Genome-wide association studies (GWASs) have identified multiple quantitative trait loci (QTLs) associated with plant architectural traits [36,37,38,39], demonstrating that cotton architecture regulation involves the interplay of multiple genes. For instance, a major locus (*PH1*) controlling plant height encoding *gibberellin 2-oxidase 1A* (*GhPH1*) suppresses cotton plant height by inactivating bioactive gibberellins (GAs) [5], while elevated endogenous GA levels induce a taller plant [40]. A synergistic regulatory module involving the GA and strigolactone (SL) pathways (GhD53-GhGARF-GhPH1/PAV^PH1^) coordinates plant height modulation [5]. Additionally, the NAC family gene *GhSBI1* was identified as a core regulator of short fruit branches, shortening internodes by repressing GA signaling [41]. The miR164-GhCUC2-GhBRC1-GhNCED1 regulatory module further controls ABA levels to shape aerial plant architecture [42]. Here, we have identified a mild *GhSP*-suppressed line, *GhSPi*-#5, with optimal plant height alongside preserved fiber yield and quality. This validates the feasibility of precise *GhSP* expression manipulation for cotton agronomic trait optimization. Notably, multiple factors, including the chromosome position, the copy number, the genetic background, and even generations, may influence the expression level of a transgene and, consequently, its functions. Considering the dose-dependent manner of *GhSP* functioning, it is necessary to continuously monitor *GhSP* expression levels and involve multiple transgenic events to create an optimal cotton germplasm for a certain genetic background or environment.

To address the challenges posed by compressed planting windows due to climatic factors in cotton-growing regions, the development of early-maturing cotton cultivars with shortened growth periods, enabling late sowing and early harvesting, has become a critical breeding objective [6]. Early-maturing varieties typically exhibit a dwarf and compact plant architecture, indicating a strong correlation between early maturity and plant architecture [43]. Recent studies have indicated that the FT-SP module plays a pivotal role in coordinating flowering time and plant architectural development. Silencing anti-florigen SP not only induces dwarfism but also accelerates flowering, a hallmark of early maturity [16,24]. However, early maturity traits are often associated with compromised fiber quality, which makes it difficult to meet the requirements for high-end textile products [6]. Knocking out *GhSP* [27], or severely suppressing *GhSP* expression levels (as in our study), results in extremely dwarfed plants with very low boll numbers and reduced fiber quality, making them unsuitable for production. Our study found that mildly suppressing *GhSP* could maintain plant height within the agronomically suitable range of 70~100 cm while significantly increasing the number of bolls per plant and reducing the rate of sterile seeds compared to extremely dwarfed plants, with fiber quality comparable to that of the wild type (Figure 2 and Figure 4). This phenotypic divergence likely stems from developmental plasticity regulated by the dose-dependent effects of *GhSP* expression. The severe suppression of *GhSP* prematurely terminates indeterminate growth in apical meristems, presumably resulting in insufficient photosynthetic source capacity to meet the sink demand of developing bolls, ultimately triggering imbalanced assimilate partitioning. In contrast, the mild suppression of *GhSP* delays the developmental transition from indeterminate to determinate growth patterns in shoot apices, thereby increasing plant biomass and maintaining the source–sink ratio within an optimized range. This study reveals the nonlinear relationship between *GhSP* expression levels and plant architecture and yield quality traits, providing new strategies for molecular design breeding in early-maturing cotton. It is proposed that utilizing tissue-specific promoters (such as those activated during the reproductive growth stage) or photoperiod-responsive elements to precisely regulate the spatiotemporal expression of *GhSP* can not only break through the architectural constraints of traditional early-maturing varieties but also avoid physiological deterioration in fiber quality.

## 5. Conclusions

In this study, RNA interference was employed to down-regulate *GhSP* expression in cotton, generating transgenic lines (*GhSPi*) with varying suppression levels. The results show that *GhSP* expression regulates cotton architecture in a dose-dependent manner. Mild suppression (such as *GhSPi*-#5, <30% reduction) resulted in a semi-dwarf phenotype (70~100 cm), with normal leaf/floral organ development and pollen fertility comparable to those of the wild type. In contrast, severe suppression (such as *GhSPi*-#14, >60% reduction) caused extreme dwarfism (20~40 cm) and the premature termination of apical meristem activity into floral buds, accompanied by reduced leaf size, floral organ malformation, and partial pollen sterility. Agronomic trait analysis revealed that *GhSPi*-#5 exhibited no significant differences from the wild type in boll size, fiber length, and yield metrics, whereas severely suppressed lines displayed a reduced boll size, compromised fiber quality, and an increased sterile seed ratio. This study demonstrates that mild *GhSP* suppression (e.g., *GhSPi*-#5) optimizes plant architecture while avoiding the detrimental effects of severe suppression on photosynthesis, reproduction, and fiber quality, providing an effective strategy for cotton architecture improvement.

## Data Availability

The data presented in this study are available on request from the corresponding author. The data are not publicly available due to privacy.

## Supplementary Materials

The following supporting information can be downloaded at: https://www.mdpi.com/article/10.3390/biology14060601/s1, Figure S1: Stem apex characteristics of *GhSPi* transgenic cotton; Figure S2: Conjoined bolls appeared in *GhSPi* transgenic lines; Table S1: Primers used in this study; Table S2: *GhSP*-RNAi transgenic cotton (#5, #8, #14) and wild-type mature fiber quality analysis.
