# Supplementary material for: RNA Interference-Mediated Suppression of GhSP (SELF-PRUNING) Modulates the Plant Architecture of Transgenic Cotton in a Dose-Dependent Manner
Source: Biology (Basel). 2025 May 25;14(6):601. doi: 10.3390/biology14060601 (PMC12189838; doi:10.3390/biology14060601)
Supplement: Supplementary file 1 [file biology-14-00601-s001.zip › biology-3646313-supplementary.pdf]

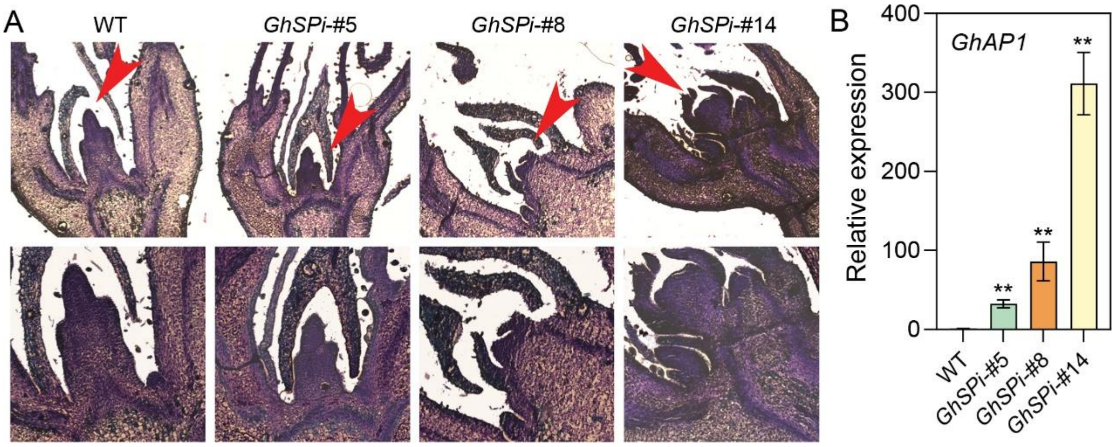

Figure S1. Stem apex characteristics of *GhSPi* transgenic cotton. (A) Histological analysis of stem tips of *GhSPi* transgenic cotton and wild-type at 14 DAS. (B) Relative expression levels of *GhAP1* in *GhSPi* transgenic cotton and wild-type at 14 DAS. Statistical significance determined by Student's t-test (n = 3): **, $p < 0.01$.

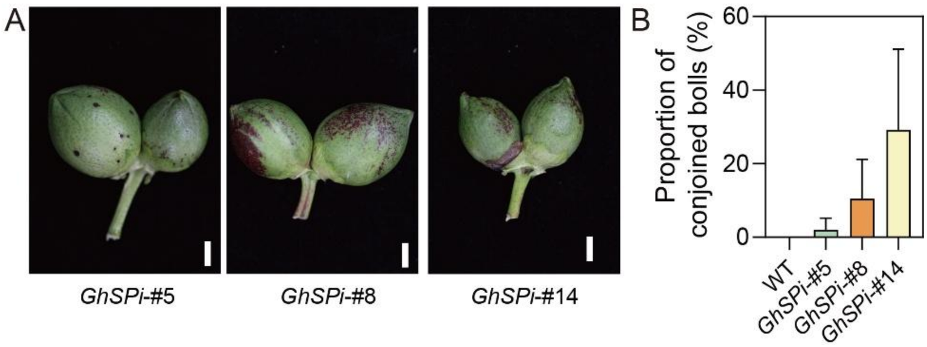

Figure S2. Conjoined bolls appeared in *GhSPi* transgenic lines. (A) Conjoined bolls of *GhSPi* transgenic plants. Scale bars, 10 mm. (B) The percentage of conjoined bolls relative to the total boll count in each transgenic line. n = 10.

Table S1 Primers used in this study

Primers	Sequences (5' to 3')	Usages
GhSPi-FP	GATTCAGATCTAGGGCAAAC	Construction of RNAi vector
GhSPi-BP	CAAGTATCTCACATGCCAAGACCCGAATTCTCCTTG	Construction of RNAi vector
GhSPi-check-F	CTTCCTGGTATCGAAACGATC	Identification of transgenic cotton
GhSPi-check-R	CCATCAAAGTTAGTCCATTTTC	Identification of transgenic cotton
RT-GhSP-F	TGAGGGAGCACTTACACTGG	RT-qPCR analysis
RT-GhSP-R	CTCACTGTTTGCCTGCCTTT	RT-qPCR analysis
RT-GhAP1-F	GCTACAGAATTTGGAACAACAGC	RT-qPCR analysis
RT-GhAP1-R	CCACCGTTTTCTCCCTTTCCTTG	RT-qPCR analysis
RT-GhACT-F	ATCCTCCGTCTTGACCTTG	RT-qPCR analysis
RT-GhACT-R	TGTCCGTCAGGCAACTCAT	RT-qPCR analysis

Table S2 *GhSP*-RNAi transgenic cotton（#5、#8、#14）and wild-type mature fiber quality analysis

	Fiber length (mm)	Uniformity Index (%)	Strength (cN/tex)	Micronaire value	Elongation ratio (%)
WT	28.20±0.22	85.37±0.19	27.07±0.29	5.13±0.05	6.47±0.05
GhSPi-#5	28.50±1.70	84.40±1.27	28.53±2.41	4.70±0.16*	6.53±0.19
GhSPi-#8	26.83±0.75*	83.73±0.33*	25.77±0.65	4.63±0.25*	6.4±0
GhSPi-#14	27.27±0.25*	81.97±0.69*	27.90±0.79	4.30±0.16*	6.43±0.05

Note: Data were obtained from three biological replicates and are expressed as the mean ± SD. Statistical significance was analyzed using a Student's t-tests (*, $p < 0.05$).
